# Some results on random smoothness

**DOI:** 10.1186/s13660-017-1386-z

**Published:** 2017-05-12

**Authors:** Xiaolin Zeng, Changying Ding

**Affiliations:** 1 School of Mathematics and Statistics, Chongqing Technology and Business University, Xuefu Road No. 21, Chongqing, 400067 P.R. China; 2 School of Mathematics and Statistics, Central South University, Xiaoxiang Middle Road No. 405, Changsha, 410083 P.R. China

**Keywords:** random normed module, random strict convexity, random smoothness, Gâteaux differentiability

## Abstract

Based on the analysis of stratification structure on random normed modules, we first present the notion of random smoothness in random normed modules. Then, we establish the relations between random smoothness and random strict convexity. Finally, a type of Gâteaux differentiability is defined for random norms, and its relation to random smoothness is given. The results are helpful in the further study of geometry of random normed modules.

## Introduction

Guo initiated a new approach to random functional analysis [1–3], whose main idea is to develop random functional analysis as functional analysis based on random normed modules, random inner product modules and random locally convex modules. In particular, since the essential applications of random functional analysis to conditional risk measures, the idea has also attracted much attention in different aspects [4–13]. Motivated by these advances, the study of geometric theory of random normed modules has begun in the direction of geometry of classical Banach spaces [14]. In [15, 16] random strict convexity and random uniform convexity are successfully introduced in random normed modules, which facilitates further study on geometrical properties of random normed modules [17]. The geometric theory of random normed modules is closely related to the geometry of Banach spaces since a complete random normed module is a kind of random generalization of a Banach space, as demonstrated by [17, 18]. From the point of view of classical Banach space theory, it is a quite natural topic to give a reasonable definition of random smoothness in random normed modules. Based on the analysis of stratification structure on random normed modules, we first present the notion of random smoothness via that of support functionals for the random closed unit ball in a random normed module. Then, the relations of random smoothness to random strict convexity are established. Finally, a type of Gâteaux differentiability equivalent to that in [19] is introduced for random norms, and its relation to random smoothness is given.

The remainder of this article is organized as follows. Section 2 is devoted to some knowledge indispensable for next two sections. Section 3 is focused on the definition and basic properties of random smoothness, where Proposition 3.4 is of vital importance in this article. As a generalization of the corresponding classical case, Proposition 3.4 is a nontrivial result which plays a key role in the proof of Proposition 3.6. Section 4 is focused on the Gâteaux differentiability of random norm, where some inequality techniques are employed in combination with stratification analysis in random normed modules to derive the main result Theorem 4.6.

Throughout this paper, we adopt the following notations [15]:

$(\Omega,{\mathcal {F}},P)$ denotes a probability space, *K* is the scalar field *R* of real numbers or *C* of complex numbers, $L^{0}({\mathcal {F}}, K)$ denotes the algebra of equivalence classes of *K*-valued ${\mathcal {F}}$-measurable random variables on Ω in the usual sense.

For any $A\in{\mathcal {F}}$, *the equivalence class of* *A*, denoted by *Ã*, is defined by $\tilde{A}=\{B\in{\mathcal {F}}:P(A\triangle B)=0\}$, where $A\triangle B$ is the symmetric difference of *A* and *B*. $P(\tilde{A})$ is defined to be $P(A)$. For two ${\mathcal {F}}$-measurable sets *G* and *D*, $G\subset D$ a.s. means $P(G\backslash D)=0$, in which case we also say $\tilde{G}\subset\tilde{D}$; $\tilde{G}\cap\tilde{D}$ denotes the equivalence class determined by $G\cap D$ and so on. For any *ξ*, $\eta\in L^{0}({\mathcal {F}},R), \xi>\eta$ means $\xi\geq\eta$ and $\xi\neq\eta$. $[\xi>\eta]$ stands for the equivalence class of the ${\mathcal {F}}$-measurable set $\{\omega\in\Omega:\xi^{0}(\omega)>\eta^{0}(\omega)\}$ (briefly, $[\xi^{0}>\eta^{0}]$), where $\xi^{0}$ and $\eta^{0}$ are arbitrarily chosen representatives of *ξ* and *η*, respectively, and $I_{[\xi>\eta]}$ stands for $\tilde{I}_{[\xi^{0}>\eta^{0}]}$. Other analogous symbols are easily understood.

Specially, $L^{0}_{+}=\{\xi\in L^{0}({\mathcal {F}},R) | \xi\geq0\}$ and $\widetilde{\mathcal {F}}$ denotes the set of equivalence classes of elements in ${\mathcal {F}}$.

## Preliminaries

In this section, we recall Hahn-Banach theorems for $L^{0}$-linear functions and a.s. bounded random linear functionals, respectively, the notion of a random normed module together with its random conjugate space, the notion of random strict convexity and frequently-used notations.

Let *E* be a left module over the algebra $L^{0}({\mathcal {F}}, K)$, a module homomorphism $f:E\rightarrow L^{0}({\mathcal {F}}, K)$ is called an $L^{0}$ (or $L^{0}({\mathcal {F}}, K)$)-linear function. If $K=R$, then a mapping $p:E\rightarrow L^{0}({\mathcal {F}},R)$ is called an $L^{0}$-sublinear function if it satisfies the following:

1. $p(\xi x)=\xi p(x)$, $\forall\xi\in L^{0}_{+}$ and $x\in E$;
2. $p(x+y)\leq p(x)+p(y),\forall x,y\in E$.

Theorem 2.1 below is an important result which will be used in the proof of Theorem 4.6.

### Theorem 2.1

[4]

*Let* *E* *be a left module over the algebra* $L^{0}({\mathcal {F}},R)$, $M\subset E$ *be an* $L^{0}({\mathcal {F}},R)$-*submodule*, $f:M\rightarrow L^{0}({\mathcal {F}},R)$ *be an* $L^{0}$-*linear function and* $p:E\rightarrow L^{0}({\mathcal {F}},R)$ *be an* $L^{0}$-*sublinear function such that* $f(x)\leq p(x),\forall x\in M$. *Then there exists an* $L^{0}$-*linear function* $g:E\rightarrow L^{0}({\mathcal {F}},R)$ *such that* *g* *extends* *f* *and* $g(x)\leq p(x),\forall x\in E$.

Definitions 2.2 and 2.3 below are fundamental notations well known in random metric theory.

### Definition 2.2

[20]

An ordered pair $(E, \Vert \cdot \Vert )$ is called a *random normed module* (briefly, an *RN* module) over *K* with base $(\Omega,{\mathcal {F}},P)$ if *E* is a left module over the algebra $L^{0}({\mathcal {F}}, K)$ and $\Vert \cdot \Vert $ is a mapping from *E* to $L^{0}_{+}$ such that the following three axioms are satisfied:

(RNM-1): $\Vert \xi x \Vert = \vert \xi \vert \Vert x \Vert , \forall\xi\in L^{0}({\mathcal {F}}, K),x\in E$;
(RNM-2): $\Vert x+y \Vert \leq \Vert x \Vert + \Vert y \Vert ,\forall x, y\in E$;
(RNM-3): $\Vert x \Vert =0$ implies $x=\theta$ (the null vector in *E*),

where $\Vert x \Vert $ is called *the random norm (or* $L^{0}$ *-norm) of the vector* *x* in *E*.

### Definition 2.3

[20]

Let $(E, \Vert \cdot \Vert )$ be an RN module. A linear operator *f* from *E* to $L^{0}({\mathcal {F}}, K)$ is an *almost surely (briefly, a.s.) bounded random linear functional* on *E* if there exists some *ξ* in $L^{0}_{+}$ such that $\vert f(x) \vert \leq\xi\cdot \Vert x \Vert , \forall x\in E$. Denote by $E^{\ast}$ the linear space of all a.s. bounded random linear functionals on *E* with the ordinary pointwise addition and scalar multiplication on linear operators. Then $(E^{\ast}, \Vert \cdot \Vert ^{\ast})$ is an RN module over *K* with base $(\Omega,{\mathcal {F}},P)$, in which the module multiplication $\cdot:L^{0}({\mathcal {F}},K)\times E^{\ast}\rightarrow E^{\ast}$ is defined by $(\xi\cdot f)(x)=\xi\cdot(f(x)), \forall\xi\in L^{0}({\mathcal {F}}, K), f\in E^{\ast}$ and $x\in E$, and the random norm $\Vert \cdot \Vert ^{\ast}: E^{\ast}\rightarrow L^{0}_{+}$ is defined by $\Vert f \Vert ^{\ast} =\wedge\{ \xi\in L^{0}_{+}: \vert f(x) \vert \leq\xi\cdot \Vert x \Vert , \forall x\in E\}, \forall f\in E^{\ast}$. $(E^{\ast}, \Vert \cdot \Vert ^{\ast})$ is called *the random conjugate space* of $(E, \Vert \cdot \Vert )$.

### Remark 2.4

In Definition 2.3, we can easily see that

1. for each *f* in $E^{\ast}$, $\vert f(x) \vert \leq \Vert f \Vert ^{\ast}\cdot \Vert x \Vert ,\forall x\in E$;
2. since $(E^{\ast}, \Vert \cdot \Vert ^{\ast})$ is also an RN module, we can have its random conjugate space $((E^{\ast})^{\ast},( \Vert \cdot \Vert ^{\ast})^{\ast})$ (briefly, $(E^{\ast\ast}, \Vert \cdot \Vert ^{\ast\ast})$). Define a mapping $J: E\rightarrow E^{\ast\ast}$ by $J(x)(f)=f(x),\forall f\in E^{\ast}$ and $x\in E$, then $\Vert J(x) \Vert ^{\ast\ast}= \Vert x \Vert $. Such a mapping *J* is called *the canonical embedding mapping* from *E* to $E^{\ast\ast}$. In particular, when *J* is surjective, we call $(E, \Vert \cdot \Vert )$ to be *random reflexive* [21].

Theorem 2.5 below is the Hahn-Banach theorem for a.s. bounded random linear functionals.

### Theorem 2.5

[4, 22]

*Let* $(E, \Vert \cdot \Vert )$ *be an RN module over* *K* *with base* $(\Omega,{\mathcal {F}},P)$, $M\subset E$ *be a linear subspace*, *and* $f:M\rightarrow L^{0}({\mathcal {F}}, K)$ *be an a*.*s*. *bounded random linear functional on* *M*. *Then there exists* $F\in E^{\ast}$ *such that* (1) $F(x)=f(x),\forall x\in M$, *and* (2) $\Vert F \Vert ^{\ast}= \Vert f \Vert ^{\ast}$. *As a consequence*, *for any* $x\in E$, *there exists* $g\in E^{\ast}$ *such that* $g(x)= \Vert x \Vert $ *and* $\Vert g \Vert ^{\ast}=I_{A_{x}}$.

Notations in Definition 2.6 below were heavily used in the study of random strict convexity and random uniform convexity [15] in order to analyze the stratification structure of *E*.

### Definition 2.6

[15]

For any $x, y$ in an RN module $(E, \Vert \cdot \Vert )$ over *K* with base $(\Omega,{\mathcal {F}},P)$, $[ \Vert x \Vert \neq0]$ is denoted by $A_{x}$, called *the support of* *x*, and we briefly write $A_{xy}=A_{x}\cap A_{y}$ and $B_{xy}=A_{x}\cap A_{y}\cap A_{x-y}$. *The random unit sphere of* *E* is defined by $S(E)=\{x\in E: \Vert x \Vert =\tilde{I}_{A}\text{ for some } A\in{\mathcal {F}} \text{ with } P(A)>0\}$. *The random closed unit ball of* *E* is defined by $U(E)=\{x\in E: \Vert x \Vert \leq1\}$.

Random strict convexity is as follows.

### Definition 2.7

[15]

An RN module $(E, \Vert \cdot \Vert )$ is said to be *random strictly convex* if for any *x* and $y\in E\backslash\{\theta\}$ such that $\Vert x+y \Vert = \Vert x \Vert + \Vert y \Vert $ there exists $\xi\in L^{0}_{+}$ such that $\xi>0$ on $A_{xy}$ and $I_{A_{xy}}x=\xi(I_{A_{xy}}y)$.

It is well known that an RN module $(E, \Vert \cdot \Vert )$ is random strictly convex if and only if for any $x,y\in S(E)$ with $P(B_{xy})>0$, $\Vert \frac{x+y}{2} \Vert <1$ on $B_{xy}$ [15], Theorem 3.1.

The notions of generalized inverse, absolute value, complex conjugate and sign of an element in $L^{0}({\mathcal {F}}, K)$ are recapitulated from [15] for Proposition 3.1 below. Let *ξ* be an element in $L^{0}({\mathcal {F}}, K)$. For an arbitrarily chosen representative $\xi^{0}$ of *ξ*, define two ${\mathcal {F}}$-random variables $(\xi^{0})^{-1}$ and $\vert \xi^{0} \vert $ respectively by

$$\bigl(\xi^{0}\bigr)^{-1}(\omega)= \textstyle\begin{cases} \frac{1}{\xi^{0}(\omega)} &\xi^{0}(\omega)\neq0, \\ 0 &\text{otherwise}, \end{cases} $$

and

$$\bigl\vert \xi^{0} \bigr\vert (\omega)= \bigl\vert \xi^{0}(\omega) \bigr\vert , \quad \forall\omega\in\Omega. $$

Then the equivalence class of $(\xi^{0})^{-1}$, denoted by $\xi^{-1}$, is called *the generalized inverse* of *ξ*; the equivalence class of $\vert \xi^{0} \vert $, denoted by $\vert \xi \vert $, is called *the absolute value* of *ξ*. When $\xi\in L^{0}({\mathcal {F}},C)$, set $\xi=u+iv$, where $u,v\in L^{0}({\mathcal {F}},R)$, $\bar{\xi}:= u-iv$ is called *the complex conjugate* of *ξ* and $\operatorname{sgn}(\xi):= \vert \xi \vert ^{-1}\cdot\xi$ is called *the sign* of *ξ*. It is obvious that $\vert \xi \vert = \vert \bar{\xi} \vert , \xi\cdot\operatorname{sgn}(\bar{\xi})= \vert \xi \vert $, $\vert \operatorname{sgn}(\xi) \vert ={I}_{A}$ and $\xi^{-1}\cdot\xi=\xi\cdot \xi^{-1}={I}_{A}$, where $A=[\xi\neq0]$.

## Random smoothness

In this section, $(E, \Vert \cdot \Vert )$ is always an RN module over *K* with base $(\Omega,{\mathcal {F}},P)$ and $(E^{\ast}, \Vert \cdot \Vert ^{\ast})$ its random conjugate space.

### Proposition 3.1

*Let* *f* *be an element in* $E^{\ast}$. *Then* $\Vert f \Vert ^{*}= \vee\{ \operatorname{Re}f(x):x\in S(E)\}$ *and* $\Vert f \Vert ^{*}= \vee\{\operatorname{Re}f(x):x\in U(E)\}$.

### Proof

It is known in [21], Proposition 2.2, that $\Vert f \Vert ^{\ast}=\vee \{ \vert f(x) \vert :x\in U(E)\}$ and in [15], Proposition 2.1, that $\Vert f \Vert ^{*}= \vee \{ \vert f(x) \vert :x\in S(E)\}$. For any $x\in S(E)$, take $x^{\prime }=\operatorname{sgn}(\overline{f(x)})x$, then $x^{\prime}\in S(E)$ and $f(x^{\prime })=\operatorname{sgn}(\overline{f(x)})f(x)= \vert f(x) \vert $, so that $\vert f(x) \vert =\operatorname{Re}f(x^{\prime })\leq\vee\{\operatorname{Re}f(x):x\in S(E)\}$, which justifies the equality $\Vert f \Vert ^{*}= \vee\{\operatorname{Re}f(x):x\in S(E)\}$. It is not difficult to see that $\Vert f \Vert ^{*}= \vee\{\operatorname{Re}f(x):x\in U(E)\}$. □

The notion of support functionals is a preparation for that of random smoothness.

### Definition 3.2

Let *A* be a subset of *E*, then a nonzero element *f* in $E^{\ast}$ is called a support functional for *A* if there exists some $x_{0}$ in *A* such that $\operatorname{Re}f(x_{0})=\vee\{\operatorname{Re}f(x):x\in A\}$, in which case $x_{0}$ is called a support point of *A*, the set $H(f,x_{0})=\{x\in E:\operatorname{Re}f(x)=\operatorname{Re}f(x_{0})\}$ is called a support hyperplane for *A*, and the support functional *f* and the support hyperplane $H(f,x_{0})$ are both said to support *A* at $x_{0}$.

### Remark 3.3

By Theorem 2.5 each point $x_{0}$ in $S(E)$ is a support point of $U(E)$ and therefore gives rise to at least one support hyperplane for $U(E)$ that supports $U(E)$ at $x_{0}$.

### Proposition 3.4

*For any* $f_{1},f_{2}\in S(E^{\ast})$ *with* $P(A_{f_{1}f_{2}})>0$ *and* $x_{0}\in S(E)$ *such that both* $f_{1}$ *and* $f_{2}$ *support* $U(E)$ *at* $x_{0}$, *namely*, $\operatorname{Re}f_{j}(x_{0})=\vee\{\operatorname{Re}f_{j}(x):x\in U(E)\}, j=1,2$ (*this time*, $\operatorname{Re}f_{j}(x_{0})= \Vert f_{j} \Vert ^{*}=I_{A_{f_{j}}},j=1,2$), *the following statements hold*:

1. *Let* $D\in\widetilde{\mathcal {F}}$ *be such that* $D\subset A_{f_{1}f_{2}}$ *and* $P(D)>0$. *If* $H(I_{D}f_{1},x_{0})=H(I_{D}f_{2},x_{0})$, *then* $H(I_{G}f_{1},x_{0})=H(I_{G}f_{2},x_{0})$ *for any* $G\in\widetilde{\mathcal {F}}$ *with* $G\subset D$ *and* $P(G)>0$;
2. *For an arbitrary* $D\in\widetilde{\mathcal {F}}$ *with* $D\subset A_{f_{1}f_{2}}$ *and* $P(D)>0$, $H(I_{D}f_{1},x_{0})=H(I_{D}f_{2},x_{0})$ *if and only if* $I_{D}f_{1}=I_{D}f_{2}$.

### Proof

(1). Let $x\in H(I_{G}f_{1},x_{0})$, then

$$\begin{aligned} \operatorname{Re}I_{D}f_{1}(I_{G}x+I_{D\backslash G}x_{0})&= \operatorname{Re}I_{D}f_{1}(I_{G}x)+\operatorname{Re}I_{D}f_{1}(I_{D\backslash G}x_{0}) \\ &= \operatorname{Re}I_{G}f_{1}(x)+\operatorname{Re}I_{D\backslash G}f_{1}(x_{0}) \\ &= \operatorname{Re}I_{G}f_{1}(x_{0})+\operatorname{Re}I_{D\backslash G}f_{1}(x_{0}) \\ &= I_{D}\operatorname{Re}f_{1}(x_{0})=\operatorname{Re}I_{D}f_{1}(x_{0}), \end{aligned}$$

so that $I_{G}x+I_{D\backslash G}x_{0}\in H(I_{D}f_{1},x_{0})=H(I_{D}f_{2},x_{0})$, which implies

$$\begin{aligned} \operatorname{Re}I_{D}f_{2}(I_{G}x+I_{D\backslash G}x_{0})=\operatorname{Re}I_{G}f_{2}(x)+\operatorname{Re}I_{D\backslash G}f_{2}(x_{0}) =\operatorname{Re}I_{D}f_{2}(x_{0}), \end{aligned}$$

thus $\operatorname{Re}I_{G}f_{2}(x)=\operatorname{Re}I_{G}f_{2}(x_{0})$, from which we can see that $H(I_{G}f_{1},x_{0})\subset H(I_{G}f_{2},x_{0})$. Similarly, $H(I_{G}f_{2},x_{0})\subset H(I_{G}f_{1},x_{0})$.

(2). (⇐) is clear.

(⇒). We only need to show that $I_{D}\operatorname{Re}f_{1}=I_{D}\operatorname{Re}f_{2}$. Assume by way of contradiction that there exists $x^{\prime}$ in *E* such that $P(D^{\prime})>0$, where $D^{\prime}=[I_{D}\operatorname{Re}f_{1}(x^{\prime})\neq I_{D}\operatorname{Re}f_{2}(x^{\prime})]\cap D$. Let

$$\begin{aligned} D_{1}^{\prime}&=\bigl[I_{D}\operatorname{Re}f_{1} \bigl(x^{\prime}\bigr)\neq0\bigr]\cap\bigl[I_{D}\operatorname{Re}f_{2} \bigl(x^{\prime }\bigr)=0\bigr]\cap D, \\ D_{2}^{\prime}&=\bigl[I_{D}\operatorname{Re}f_{1} \bigl(x^{\prime}\bigr)=0\bigr]\cap\bigl[I_{D}\operatorname{Re}f_{2} \bigl(x^{\prime }\bigr)\neq0\bigr]\cap D, \\ D_{3}^{\prime}&=\bigl[I_{D}\operatorname{Re}f_{1} \bigl(x^{\prime}\bigr)\neq0\bigr]\cap\bigl[I_{D}\operatorname{Re}f_{2} \bigl(x^{\prime }\bigr)\neq0\bigr]\cap\bigl[I_{D}\operatorname{Re}f_{1} \bigl(x^{\prime}\bigr)\neq I_{D}\operatorname{Re}f_{2} \bigl(x^{\prime}\bigr)\bigr]\cap D, \end{aligned}$$

then $D^{\prime}=D_{1}^{\prime}\cup D_{2}^{\prime}\cup D_{3}^{\prime}$. It is enough to show that $P(D^{\prime})=0$.

If $P(D_{1}^{\prime})>0$, let $x_{1}^{\prime}=I_{D_{1}^{\prime }}(\operatorname{Re}f_{1}(x^{\prime}))^{-1}x^{\prime}$, then

$$\operatorname{Re}f_{1}\bigl(x_{1}^{\prime}\bigr)=I_{D_{1}^{\prime}} \bigl(\operatorname{Re}f_{1}\bigl(x^{\prime }\bigr)\bigr)^{-1}\operatorname{Re}f_{1} \bigl(x^{\prime}\bigr)=I_{D_{1}^{\prime}}=I_{D_{1}^{\prime }}I_{A_{f_{1}}}=I_{D_{1}^{\prime}}\operatorname{Re}f_{1}(x_{0}), $$

which implies that $x_{1}^{\prime}\in H(I_{D_{1}^{\prime}}f_{1},x_{0})$. Since $H(I_{D}f_{1},x_{0})=H(I_{D}f_{2},x_{0})$ and $D_{1}^{\prime}\subset D$, by (1) of this proposition we have $x_{1}^{\prime}\in H(I_{D_{1}^{\prime }}f_{2},x_{0})$, namely, $\operatorname{Re}I_{D_{1}^{\prime}}f_{2}(x_{1}^{\prime })=\operatorname{Re}I_{D_{1}^{\prime}}f_{2}(x_{0})=I_{D_{1}^{\prime}}$. On the other hand, $\operatorname{Re}I_{D_{1}^{\prime}}f_{2}(x_{1}^{\prime}) =I_{D_{1}^{\prime }}\operatorname{Re}f_{2}(x_{1}^{\prime}) =I_{D_{1}^{\prime}}(\operatorname{Re}f_{1}(x^{\prime }))^{-1}\operatorname{Re}f_{2}(x^{\prime}) =0$. The contradiction shows that $P(D_{1}^{\prime})=0$.

Similarly, $P(D_{2}^{\prime})=0$.

Now let us show that $P(D_{3}^{\prime})=0$ as follows.

If $P(D_{3}^{\prime})>0$, then let $y=I_{D_{3}^{\prime}}(\operatorname{Re}f_{1}(x^{\prime }))^{-1}x^{\prime}$. It is easy to see that $\operatorname{Re}f_{1}(y)=I_{D_{3}^{\prime }}(\operatorname{Re}f_{1}(x^{\prime}))^{-1}\times \operatorname{Re}f_{1}(x^{\prime})=I_{D_{3}^{\prime}}$, which implies that $\operatorname{Re}I_{D_{3}^{\prime}}f_{1}(y)=I_{D_{3}^{\prime }}=I_{D_{3}^{\prime}}I_{A_{f_{1}}}=\operatorname{Re}I_{D_{3}^{\prime}}f_{1}(x_{0})$, namely, $y\in H(I_{D_{3}^{\prime}}f_{1},x_{0})$. Again by (1) of this proposition we have $H(I_{D_{3}^{\prime}}f_{1},x_{0})=H(I_{D_{3}^{\prime}}f_{2},x_{0})$. Consequently, $\operatorname{Re}I_{D_{3}^{\prime}}f_{2}(y)=\operatorname{Re}I_{D_{3}^{\prime }}f_{2}(x_{0})=I_{D_{3}^{\prime}}$. On the other hand, by the definition of *y* and $D_{3}^{\prime}$ we have that $\operatorname{Re}I_{D_{3}^{\prime }}f_{2}(y)=\operatorname{Re}I_{D_{3}^{\prime}}f_{2}(I_{D_{3}^{\prime}}(\operatorname{Re}f_{1}(x^{\prime }))^{-1}x^{\prime})=\operatorname{Re}I_{D_{3}^{\prime}}(\operatorname{Re}f_{1}(x^{\prime }))^{-1}f_{2}(x^{\prime})=I_{D_{3}^{\prime}}(\operatorname{Re}f_{1}(x^{\prime }))^{-1}\operatorname{Re}f_{2}(x^{\prime})\neq1$ on $D_{3}^{\prime}$, which contradicts the fact that

$$\operatorname{Re}I_{D_{3}^{\prime}}f_{2}(y)=\operatorname{Re}I_{D_{3}^{\prime}}f_{2}(x_{0})=I_{D_{3}^{\prime}}. $$

Thus $P(D_{3}^{\prime})=0$.

Therefore, $P(D^{\prime})=0$ and this completes the proof. □

### Definition 3.5

In an RN module $(E, \Vert \cdot \Vert )$, an element $x_{0}$ of $S(E)$ is called a point of random smoothness of $U(E)$ if $H(I_{A_{f_{1}f_{2}}}f_{1},x_{0})=H(I_{A_{f_{1}f_{2}}}f_{2},x_{0})$ for any two support functionals $f_{1},f_{2}$ for $U(E)$ at $x_{0}$ with $P(A_{f_{1}f_{2}})>0$. *E* is said to be random smooth if each point of $S(E)$ is a point of random smoothness of $U(E)$.

### Proposition 3.6

*Suppose that* *E* *is an RN module and that* $x_{0}\in S(E)$. *Then the following are equivalent*.

1. $x_{0}$ *is a point of random smoothness of* $U(E)$;
2. *If both* $f_{1}$ *and* $f_{2}$ *in* $S(E^{\ast})$ *support* $U(E)$ *at* $x_{0}$ *and* $P(A_{f_{1}f_{2}})>0$, *then* $I_{A_{f_{1}f_{2}}}f_{1}=I_{A_{f_{1}f_{2}}}f_{2}$;
3. *If* $f_{1}$ *and* $f_{2}$ *in* $S(E^{\ast})$ *satisfy* $\operatorname{Re}I_{A_{f_{1}f_{2}}}f_{j}(x_{0})=I_{A_{f_{1}f_{2}}}(j=1,2)$, *then* $I_{A_{f_{1}f_{2}}}f_{1}=I_{A_{f_{1}f_{2}}}f_{2}$;
4. *If* $f_{1}$ *and* $f_{2}$ *in* $S(E^{\ast})$ *satisfy* $f_{1}(x_{0})=f_{2}(x_{0})=I_{A_{f_{1}f_{2}}}$, *then* $I_{A_{f_{1}f_{2}}}f_{1}=I_{A_{f_{1}f_{2}}}f_{2}$.

### Proof

(1)⇒(2). Assume that $I_{A_{f_{1}f_{2}}}f_{1}\neq I_{A_{f_{1}f_{2}}}f_{2}$, then $I_{A_{f_{1}f_{2}}} \Vert f_{1}-f_{2} \Vert ^{\ast}\neq0$. It is easy to see that $P(D)>0$, where $D=A_{f_{1}f_{2}}\cap[ \Vert f_{1}-f_{2} \Vert ^{\ast}\neq0]$. Considering $I_{D}f_{1},I_{D}f_{2}\in S(E^{\ast})$ and $I_{D}x_{0}\in S(E)$, we can gte $\operatorname{Re}I_{D}f_{j}(x_{0})=I_{D}\operatorname{Re}f_{j}(x_{0})=I_{D}\cdot\vee\{\operatorname{Re}f_{j}(x):x\in U(E)\}=\vee \{\operatorname{Re}I_{D}f_{j}(x):x\in U(E)\}$ ($j=1,2$). Namely, both $I_{D}f_{1}$ and $I_{D}f_{2}$ are support functionals for $U(E)$ at $x_{0}$. By Definition 3.5 we have $H(I_{D}f_{1},x_{0})=H(I_{D}f_{2},x_{0})$, and then by Proposition 3.4(2) $I_{D}f_{1}=I_{D}f_{2}$, which leads to $I_{D} \Vert f_{1}-f_{2} \Vert ^{\ast}=0$, a contradiction.

(2)⇒(3). Observing that $\operatorname{Re}I_{A_{f_{1}f_{2}}}f_{j}(x_{0})=I_{A_{f_{1}f_{2}}}= \Vert I_{A_{f_{1}f_{2}}}f_{j} \Vert ^{\ast }=\vee\{\operatorname{Re}I_{A_{f_{1}f_{2}}}f_{j}(x):x\in U(E)\} (j=1,2)$, namely, both $I_{A_{f_{1}f_{2}}}f_{1}$ and $I_{A_{f_{1}f_{2}}}f_{2}$ support $U(E)$ at $x_{0}$, we obtain that $I_{A_{f_{1}f_{2}}}f_{1}=I_{A_{f_{1}f_{2}}}f_{2}$ by (2).

(3)⇒(4) is obvious.

(4)⇒(1) Suppose that $f_{1},f_{2}\in E^{\ast}$ are two support functionals for $U(E)$ with $P(A_{f_{1}f_{2}})>0$, namely,

$$\operatorname{Re}f_{j}(x_{0})=\vee\bigl\{ \operatorname{Re}f_{j}(x):x\in U(E) \bigr\} = \Vert f_{j} \Vert ^{\ast}\quad (j=1,2). $$

It is enough to show that $H(I_{A_{f_{1}f_{2}}}f_{1},x_{0})=H(I_{A_{f_{1}f_{2}}}f_{2},x_{0})$ by Proposition 3.4(2). Clearly,

$$\begin{aligned} &\operatorname{Re}I_{A_{f_{1}f_{2}}}\bigl( \Vert f_{1} \Vert ^{\ast} \bigr)^{-1}f_{1}(x_{0})=\operatorname{Re}I_{A_{f_{1}f_{2}}}\bigl( \Vert f_{2} \Vert ^{\ast}\bigr)^{-1}f_{2}(x_{0})=I_{A_{f_{1}f_{2}}}, \\ &\quad I_{A_{f_{1}f_{2}}}\bigl( \Vert f_{1} \Vert ^{\ast } \bigr)^{-1}f_{1},I_{A_{f_{1}f_{2}}}\bigl( \Vert f_{2} \Vert ^{\ast}\bigr)^{-1}f_{2}\in S \bigl(E^{\ast}\bigr). \end{aligned}$$

From

$$\Vert f_{1} \Vert ^{\ast}=\operatorname{Re}f_{1}(x_{0}) \leq \bigl\vert f_{1}(x_{0}) \bigr\vert \leq \Vert f_{1} \Vert ^{\ast} \Vert x_{0} \Vert = \Vert f_{1} \Vert ^{\ast}\cdot I_{A_{x_{0}}}\leq \Vert f_{1} \Vert ^{\ast} $$

it follows that $\vert f_{1}(x_{0}) \vert = \Vert f_{1} \Vert ^{\ast}$, namely, $\vert \operatorname{Re}f_{1}(x_{0})-i\operatorname{Re}f_{1}(ix_{0}) \vert = \Vert f_{1} \Vert ^{\ast}$, which together with $\operatorname{Re}f_{1}(x_{0})= \Vert f_{1} \Vert ^{\ast}$ implies that $\operatorname{Re}f_{1}(ix_{0})=0$; consequently, $f_{1}(x_{0})=\operatorname{Re}f_{1}(x_{0})= \Vert f_{1} \Vert ^{\ast}$. In the same way, $f_{2}(x_{0})= \Vert f_{2} \Vert ^{\ast}$. Thus $I_{A_{f_{1}f_{2}}}( \Vert f_{1} \Vert ^{\ast })^{-1}f_{1}(x_{0})=I_{A_{f_{1}f_{2}}}( \Vert f_{2} \Vert ^{\ast })^{-1}f_{2}(x_{0})=I_{A_{f_{1}f_{2}}}$. Then by (4) $I_{A_{f_{1}f_{2}}}( \Vert f_{1} \Vert ^{\ast})^{-1}f_{1}=I_{A_{f_{1}f_{2}}}( \Vert f_{2} \Vert ^{\ast})^{-1}f_{2}$. Therefore, $H(I_{A_{f_{1}f_{2}}}f_{1},x_{0})=H(I_{A_{f_{1}f_{2}}}f_{2},x_{0})$. □

### Corollary 3.7

*Suppose that* *E* *is an RN module and that* $x_{0}\in S(E)$. *Then the following are equivalent*.

1. *E* *is random smooth*;
2. *For each* $x\in S(E)$, *if both* $f_{1}$ *and* $f_{2}$ *in* $S(E^{\ast})$ *support* $U(E)$ *at* *x* *with* $P(A_{f_{1}f_{2}})>0$, *then* $I_{A_{f_{1}f_{2}}}f_{1}=I_{A_{f_{1}f_{2}}}f_{2}$;
3. *For each* $x\in S(E)$, *if* $f_{1}$ *and* $f_{2}$ *in* $S(E^{\ast})$ *satisfy* $\operatorname{Re}I_{A_{f_{1}f_{2}}}f_{1}(x)=\operatorname{Re}I_{A_{f_{1}f_{2}}}f_{2}(x)=I_{A_{f_{1}f_{2}}}$, *then* $I_{A_{f_{1}f_{2}}}f_{1}=I_{A_{f_{1}f_{2}}}f_{2}$;
4. *For each* $x\in S(E)$, *if* $f_{1}$ *and* $f_{2}$ *in* $S(E^{\ast})$ *satisfy* $I_{A_{f_{1}f_{2}}}f_{1}(x)=I_{A_{f_{1}f_{2}}}f_{2}(x)=I_{A_{f_{1}f_{2}}}$, *then* $I_{A_{f_{1}f_{2}}}f_{1}=I_{A_{f_{1}f_{2}}}f_{2}$.

The relations between random smoothness and random strict convexity are described by Theorems 3.8 and 3.9 below.

### Theorem 3.8

*Suppose that* *E* *is an RN module*. *If* $E^{\ast}$ *is random strictly convex*, *then* *E* *is random smooth*.

### Proof

Suppose that *E* is not random smooth, by Corollary 3.7, there exist $x\in S(E)$ and $f_{1},f_{2}\in S(E^{\ast})$ such that

1 $$\begin{aligned} I_{A_{f_{1}f_{2}}}f_{1}(x)=I_{A_{f_{1}f_{2}}}f_{2}(x)=I_{A_{f_{1}f_{2}}} \end{aligned}$$

and

2 $$\begin{aligned} I_{A_{f_{1}f_{2}}}f_{1}\neq I_{A_{f_{1}f_{2}}}f_{2}. \end{aligned}$$

From (1) it follows that

$$\frac{1}{2}I_{A_{f_{1}f_{2}}}(f_{1}+f_{2}) (x)=I_{A_{f_{1}f_{2}}}, $$

which yields

$$\biggl\Vert \frac{1}{2}I_{A_{f_{1}f_{2}}}(f_{1}+f_{2}) \biggr\Vert =I_{A_{f_{1}f_{2}}}, $$

namely,

$$\Vert I_{A_{f_{1}f_{2}}}f_{1}+I_{A_{f_{1}f_{2}}}f_{2} \Vert =2I_{A_{f_{1}f_{2}}}= \Vert I_{A_{f_{1}f_{2}}}f_{1} \Vert + \Vert I_{A_{f_{1}f_{2}}}f_{2} \Vert . $$

By the random strict convexity of $E^{\ast}$, there exists $\xi\in L^{0}_{+}$ such that $\xi>0$ on $A_{f_{1}f_{2}}$ and $I_{A_{f_{1}f_{2}}}f_{1}=\xi I_{A_{f_{1}f_{2}}}f_{2}$, so that $I_{A_{f_{1}f_{2}}} \Vert f_{1} \Vert =\xi I_{A_{f_{1}f_{2}}} \Vert f_{2} \Vert $, which implies $\xi I_{A_{f_{1}f_{2}}}=I_{A_{f_{1}f_{2}}}$, thus $I_{A_{f_{1}f_{2}}}f_{1}=I_{A_{f_{1}f_{2}}}f_{2}$, a contradiction to (2). □

### Theorem 3.9

*Suppose that* *E* *is an RN module*. *If* $E^{\ast}$ *is random smooth*, *then* *E* *is random strictly convex*.

### Proof

Assume by way of contradiction that *E* is not random strictly convex, then there exist $x_{1},x_{2}\in S(E)$ and $D\in \widetilde{\mathcal {F}}$ with $D\subset B_{x_{1}x_{2}}$ and $P(D)>0$ such that

$$I_{D} \biggl\Vert \frac{x_{1}+x_{2}}{2} \biggr\Vert =I_{D}. $$

By Theorem 2.5, there exists $f_{0}\in E^{\ast}$ such that

$$f_{0} \biggl(I_{D}\frac{x_{1}+x_{2}}{2} \biggr)=I_{D} \biggl\Vert \frac {x_{1}+x_{2}}{2} \biggr\Vert =I_{D} $$

and $\Vert f_{0} \Vert ^{\ast}=I_{D}$. Noticing that

3 $$\begin{aligned} I_{D}=\frac{I_{D}}{2}f_{0}(x_{1})+ \frac{I_{D}}{2}f_{0}(x_{2})\leq\frac {I_{D}}{2} \bigl\vert f_{0}(x_{1}) \bigr\vert +\frac{I_{D}}{2} \bigl\vert f_{0}(x_{2}) \bigr\vert \leq I_{D}, \end{aligned}$$

we have

$$\frac{I_{D}}{2} \bigl\vert f_{0}(x_{1}) \bigr\vert + \frac{I_{D}}{2} \bigl\vert f_{0}(x_{2}) \bigr\vert =I_{D}, $$

where $\frac{I_{D}}{2} \vert f_{0}(x_{1}) \vert \leq\frac{1}{2} I_{D}$ and $\frac {I_{D}}{2} \vert f_{0}(x_{2}) \vert \leq\frac{1}{2} I_{D}$. Consequently, $I_{D} \vert f_{0}(x_{i}) \vert =I_{D} (i=1,2)$. By (3) $I_{D}=\frac{I_{D}}{2}\operatorname{Re}f_{0}(x_{1})+\frac {I_{D}}{2}\operatorname{Re}f_{0}(x_{2})$, which in combination with $I_{D} \vert f_{0}(x_{1}) \vert =I_{D} \vert f_{0}(x_{2}) \vert =I_{D}$ yields $\operatorname{Re}f_{0}(x_{1})=\operatorname{Re}f_{0}(x_{2})=I_{D}$, and hence $I_{D}f_{0}(x_{1})=I_{D}f_{0}(x_{2})=I_{D}$. Using the canonical embedding mapping (see Remark 2.4), we have $J(I_{D}x_{1})(f_{0})=f_{0}(I_{D}x_{1})=f_{0}(I_{D}x_{2})=J(I_{D}x_{2})(f_{0})=I_{D}$, thus $\Vert J(I_{D}x_{j}) \Vert ^{\ast\ast}=\vee\{J(I_{D}x_{j})(f):f\in U(E^{\ast})\} =I_{D}=J(I_{D}x_{j})(f_{0})\ (j=1,2)$, namely, both $J(I_{D}x_{1})$ and $J(I_{D}x_{2})$ support $U(E^{\ast})$ at $f_{0}$. Since $E^{\ast}$ is random smooth, by Corollary 3.7 we can obtain that $I_{D}J(I_{D}x_{1})=I_{D}J(I_{D}x_{2})$ so that $I_{D} \Vert J(I_{D}x_{1}-I_{D}x_{2}) \Vert ^{\ast\ast }=0$, which yields $I_{D} \Vert x_{1}-x_{2} \Vert =0$, a contradiction. □

### Proposition 3.10

*Let* *E* *be a random reflexive RN module*. *Then the following hold*.

1. *E* *is random strictly convex if and only if* $E^{\ast}$ *is random smooth*;
2. *E* *is random smooth if and only if* $E^{\ast}$ *is random strictly convex*.

### Proof

Since the canonical embedding mapping *J* is random-norm preserving and linear, and $J(E)=E^{\ast\ast}$, it is a straightforward verification by Theorems 3.8 and 3.9. □

## Gâteaux differentiability of random norm

In classical normed spaces the function $t\mapsto( \Vert x_{0}+ty \Vert - \Vert x_{0} \Vert )/t$ from $R\backslash\{0\}$ to *R* plays an important role in Gâteaux differentiability of the classical norm [23]. Motivated by this, we consider in an RN module $(E, \Vert \cdot \Vert )$ the mapping *f* defined by

$$f(t,y)=\frac{ \Vert x_{0}+ty \Vert - \Vert x_{0} \Vert }{t},\quad \forall t\in{L^{0}(\mathcal {F},R)}\backslash \{0\},\forall y\in E, $$

where $x_{0}$ is a fixed element in $S(E)$. It is feasible to define the Gâteaux differentiability of random norm via $f(t,y)$ and establish its relation to random smoothness. It should be pointed out that some terminologies and properties in this section are closely related to [19], Section 5.

For any $D\in\widetilde{\mathcal {F}}$ with $P(D)>0$ and $t_{1},t_{2}$ in ${L^{0}(\mathcal {F},R)}$ such that $0< t_{1}< t_{2}$ on *D*, we can verify that

$$\begin{aligned} &I_{D}\frac{ \Vert x_{0}+t_{1}y \Vert - \Vert x_{0} \Vert }{t_{1}}\leq I_{D}\frac{ \Vert x_{0}+t_{2}y \Vert - \Vert x_{0} \Vert }{t_{2}}, \\ &I_{D}\frac{ \Vert x_{0}-t_{2}y \Vert - \Vert x_{0} \Vert }{-t_{2}}\leq I_{D}\frac{ \Vert x_{0}-t_{1}y \Vert - \Vert x_{0} \Vert }{-t_{1}} \end{aligned}$$

and

$$I_{D}\frac{ \Vert x_{0}-t_{1}y \Vert - \Vert x_{0} \Vert }{-t_{1}}\leq I_{D}\frac{ \Vert x_{0}+t_{1}y \Vert - \Vert x_{0} \Vert }{t_{1}} $$

as in the classical case, see [23] for details. Consequently, $f(t,y)$ is nondecreasing in *t* in the sense that $I_{D}f(t_{1},y)\leq I_{D}f(t_{2},y)$ for any $t_{1},t_{2}\in{L^{0}(\mathcal {F},R)}$ and $D\in\widetilde{\mathcal {F}}$ with $P(D)>0$ such that $t_{1},t_{2}\neq0$ on *D* and $t_{1}< t_{2}$ on *D*. Besides, $f(t,y)$ possesses the following properties as described in Lemma 4.1.

Lemmas 4.1(1) and 4.2 below are implied by [19], Lemma 5.1, and [19], Theorem 5.5(1), respectively. Here we present slightly different proofs to illustrate the typical stratification analysis on random normed modules and to keep self-contained.

### Lemma 4.1

(1) $\wedge\{I_{D}f(t,y):t\in L^{0}({\mathcal {F}},R),t>0\textit{ on }D\}=I_{D}\cdot\wedge\{f(s,y):s\in R,s>0\}$;

(2) $\wedge\{I_{D}f(\xi s,y):s\in R,s>0\}=I_{D}\cdot\wedge\{ f(s,y):s\in R,s>0\},\forall\xi\in L^{0}({\mathcal {F}},R),\xi>0$ *on* *D*;

(3) $\vee\{I_{D}f(t,y):t\in L^{0}({\mathcal {F}},R),t<0\textit{ on }D\}=I_{D}\cdot\vee\{f(s,y):s\in R,s<0\}$.

### Proof

(1). It is clear that

$$\wedge\bigl\{ I_{D}f(t,y):t\in L^{0}({\mathcal {F}},R),t>0 \text{ on }D\bigr\} \leq I_{D}\cdot\wedge\bigl\{ f(s,y):s\in R,s>0\bigr\} . $$

For any fixed $t\in L^{0}({\mathcal {F}},R)$ with $t>0\text{ on }D$, denote $D_{0}(t)=D\cap[t\geq1]$ and $D_{n}(t)=D\cap[\frac{1}{n+1}\leq t<\frac{1}{n}],\forall n\geq1$, then $D=\sum_{n=0}^{\infty}D_{n}(t)$. Since

$$I_{D_{n}(t)}f(t,y)\geq I_{D_{n}(t)}f\biggl(\frac{1}{n+1},y\biggr) \geq I_{D_{n}(t)}\cdot \wedge\bigl\{ f(s,y):s\in R,s>0\bigr\} ,\quad \forall n, $$

we obtain that

$$\begin{aligned} I_{D}f(t,y)&=\sum_{n=0}^{\infty}I_{D_{n}(t)}f(t,y) \geq\sum_{n=0}^{\infty}I_{D_{n}(t)}\cdot \wedge\bigl\{ f(s,y):s\in R,s>0\bigr\} \\ &= I_{D}\cdot\wedge\bigl\{ f(s,y):s\in R,s>0\bigr\} . \end{aligned}$$

The proofs of (2) and (3) are similar. □

### Lemma 4.2

*Denote* $G_{+}(x_{0},y)=\wedge\{f(t,y):t\in R,t>0\},G_{-}(x_{0},y)=\vee\{ f(t,y):t\in R,t<0\}$. *Then* $G_{+}(x_{0},y)$ *and* $G_{-}(x_{0},y)$ *satisfy the following*:

1. $G_{+}(x_{0},\xi y)=\xi G_{+}(x_{0},y),G_{-}(x_{0},\xi y)=\xi G_{-}(x_{0},y)$;
2. $G_{+}(x_{0},y_{1}+y_{2})\leq G_{+}(x_{0},y_{1})+G_{+}(x_{0},y_{2})$,

*where* $x_{0}\in S(E),y,y_{1},y_{2}\in E,\xi\in L^{0}_{+}$.

### Proof

(1). For any $\xi\in L^{0}_{+}$, let $A=[\xi>0]$, then by Lemma 4.1

$$\begin{aligned} G_{+}(x_{0},\xi y)&= \wedge \bigl\{ f(t,\xi y):t\in R,t>0 \bigr\} \\ &=\wedge \biggl\{ \frac{ \Vert x_{0}+t\xi y \Vert - \Vert x_{0} \Vert }{t}:t\in R,t>0 \biggr\} \\ &=\wedge \biggl\{ \frac{I_{A} \Vert x_{0}+t\xi y \Vert -I_{A} \Vert x_{0} \Vert }{t}:t\in R,t>0 \biggr\} \\ &= I_{A}\xi\cdot\wedge \biggl\{ I_{A}\cdot \frac{ \Vert x_{0}+t\xi y \Vert - \Vert x_{0} \Vert }{t\xi}:t\in R,t>0 \biggr\} \\ &= I_{A}\xi\cdot\wedge \biggl\{ I_{A}\cdot \frac{ \Vert x_{0}+t y \Vert - \Vert x_{0} \Vert }{t}:t\in R,t>0 \biggr\} \\ &= I_{A}\xi G_{+}(x_{0},y) \\ &= \xi G_{+}(x_{0},y). \end{aligned}$$

The proof of the other equality is similar.

(2). Notice that

4 $$\begin{aligned} & \wedge\bigl\{ f(2t,y_{1}):t\in R,t>0\bigr\} +\wedge\bigl\{ f(2t,y_{2}):t\in R,t>0\bigr\} \\ &\quad =\wedge\bigl\{ f(2t,y_{1})+f(2t,y_{2}):t\in R,t>0\bigr\} . \end{aligned}$$

In fact, for any positive numbers $t_{1}$ and $t_{2}$,

$$\begin{aligned} f(2t_{1},y_{1})+f(2t_{2},y_{2})& \geq \bigl[f(2t_{1},y_{1})+f(2t_{1},y_{2}) \bigr]\wedge \bigl[f(2t_{2},y_{1})+f(2t_{2},y_{2}) \bigr] \\ &\geq \wedge\bigl\{ f(2t,y_{1})+f(2t,y_{2}):t\in R,t>0\bigr\} . \end{aligned}$$

Consequently, the left-hand side of (4) is not less than the right-hand side, so that (4) can be easily verified. Then, by the following

$$\begin{aligned} f(t,y_{1}+y_{2})&= \frac{ \Vert x_{0}+t(y_{1}+y_{2}) \Vert - \Vert x_{0} \Vert }{t} \\ &\leq \frac{ \Vert x_{0}+2ty_{1} \Vert - \Vert x_{0} \Vert }{2t}+\frac{ \Vert x_{0}+2ty_{2} \Vert - \Vert x_{0} \Vert }{2t} \\ &= f(2t,y_{1})+f(2t,y_{2})\quad (\forall t\in R,t>0) \end{aligned}$$

it is easy to see that

$$\begin{aligned} &\wedge\bigl\{ f(t,y_{1}+y_{2}):t\in R,t>0\bigr\} \\ &\quad \leq\wedge \bigl\{ f(2t,y_{1})+f(2t,y_{2}):t\in R,t>0\bigr\} \\ &\quad =\wedge\bigl\{ f(2t,y_{1}):t\in R,t>0\bigr\} +\wedge\bigl\{ f(2t,y_{2}):t\in R,t>0\bigr\} , \end{aligned}$$

which implies that

$$G_{+}(x_{0},y_{1}+y_{2})\leq G_{+}(x_{0},y_{1})+G_{+}(x_{0},y_{2}). $$

 □

The Gâteaux differentiability was defined for proper local functions from a random locally convex module to $\bar{L}^{0}(\mathcal {F})$ in [19], Definition 5.2. Since a random locally convex module is a generalization of a random normed module and a random norm is ${L}^{0}$-convex, it is easy to see that a random norm is also a proper local function. For random norms we present the following definition of Gâteaux differentiability, which is equivalent to that in [19], Definition 5.2. Under Definition 4.3 we can establish the relations among supporting functionals, points of random smoothness and Gâteaux differentiability of random norms.

### Definition 4.3

Let $(E, \Vert \cdot \Vert )$ be an RN module, $x_{0}\in S(E)$ and $y\in E$. Then $G_{-}(x_{0},y)$ and $G_{+}(x_{0},y)$ are respectively called the left-hand and right-hand Gâteaux derivative of the random norm $\Vert \cdot \Vert $ at $x_{0}$ in the direction y. $\Vert \cdot \Vert $ is called Gâteaux differentiable at $x_{0}$ in the direction *y* if $G_{-}(x_{0},y)=G_{+}(x_{0},y)$, in which case the common value of $G_{-}(x_{0},y)$ and $G_{+}(x_{0},y)$ is denoted by $G(x_{0},y)$ and is called the Gâteaux derivative of $\Vert \cdot \Vert $ at $x_{0}$ in the direction *y*. If $\Vert \cdot \Vert $ is Gâteaux differentiable at $x_{0}$ in every direction $y\in E$, then it is called Gâteaux differentiable at $x_{0}$. Finally, if $\Vert \cdot \Vert $ is Gâteaux differentiable at every point of the random unit sphere $S(E)$, then $\Vert \cdot \Vert $ is said to be Gâteaux differentiable.

### Remark 4.4

It is obvious that $G_{-}(x,y)\leq G_{+}(x,y), G_{-}(x,-y)=-G_{+}(x,y)$ and $G_{-}(x,x)=G_{+}(x,x)=G(x,x)= \Vert x \Vert =I_{A_{x}}$ for any *x* in $S(E)$ and *y* in *E*.

### Lemma 4.5

*Let* $(E, \Vert \cdot \Vert )$ *be an RN module*, $x_{0}\in S(E)$ *and* $f\in S(E^{\ast })$ *such that* $P(D)>0$, *where* $D=A_{x_{0}}\cap A_{f}$. *Then* $I_{D}f$ *supports* $U(E)$ *at* $x_{0}$ *if and only if*

5 $$ I_{D}G_{-}(x_{0},y)\leq I_{D}\operatorname{Re}f(y) \leq I_{D}G_{+}(x_{0},y),\quad\forall y\in E. $$

### Proof

(⇒). By the following facts

$$\begin{aligned} &\operatorname{Re}I_{D}f(x_{0})=\vee\bigl\{ \operatorname{Re}I_{D}f(x):x\in U(E) \bigr\} =I_{D} \Vert f \Vert ^{\ast}=I_{D}, \\ &I_{D}f(x_{0})=\operatorname{Re}I_{D}f(x_{0})-i\operatorname{Re}I_{D}f(ix_{0}) \quad\text{and}\quad I_{D}f(x_{0})\leq I_{D} \Vert f \Vert ^{\ast} \Vert x_{0} \Vert =I_{D}, \end{aligned}$$

one can obtain that $I_{D}f(x_{0})=I_{D}$. Since

$$I_{D}\operatorname{Re}f(ty)=I_{D}\operatorname{Re}f(x_{0}+ty)-I_{D}\operatorname{Re}f(x_{0}) \leq I_{D}\bigl( \Vert x_{0}+ty \Vert - \Vert x_{0} \Vert \bigr) $$

for any positive numbers *t* and any $y\in E$, the following inequalities hold:

$$\begin{aligned} I_{D}\frac{ \Vert x_{0}-ty \Vert - \Vert x_{0} \Vert }{-t}&=-I_{D}\frac{ \Vert x_{0}+t(-y) \Vert - \Vert x_{0} \Vert }{t} \\ &\leq-I_{D}\frac{\operatorname{Re}f(t(-y))}{t} =I_{D} \operatorname{Re}f(y)\leq I_{D}\frac{ \Vert x_{0}+ty \Vert - \Vert x_{0} \Vert }{t}, \end{aligned}$$

which implies (5).

(⇐). Since $G_{-}(x_{0},x_{0})= \Vert x_{0} \Vert =G_{+}(x_{0},x_{0})$, it follows that $I_{D}\operatorname{Re}f(x_{0})=I_{D}$ by (5). Noticing that

$$\operatorname{Re}I_{D}f(x_{0})=I_{D}=\vee\bigl\{ \operatorname{Re}I_{D}f(x):x\in U(E)\bigr\} , $$

we complete the proof. □

### Theorem 4.6

*Let* $(E, \Vert \cdot \Vert )$ *be an RN module and* $x_{0}$ *be a point in* $S(E)$. *Then the following statements hold*:

1. $x_{0}$ *is a point of random smoothness of* $U(E)$ *if and only if* $\Vert \cdot \Vert $ *is Gâteaux differentiable at* $x_{0}$;
2. *If* $x_{0}$ *is a point of random smoothness of* $U(E)$ *and* *f* *in* $S(E^{\ast})$ *supports* $U(E)$ *at* $x_{0}$, *then* $I_{A_{f}}G(x_{0},y)=\operatorname{Re}I_{A_{f}}f(y)$.

### Proof

(1). (⇒). Assume by way of contradiction that $\Vert \cdot \Vert $ is not Gâteaux differentiable at $x_{0}$. Then there exists $y_{0}$ in *E* such that $G_{-}(x_{0},y_{0})< G_{+}(x_{0},y_{0})$, namely, $P(M)>0$, where $M=[G_{-}(x_{0},y_{0})< G_{+}(x_{0},y_{0})]$. Since $I_{A_{x_{0}}^{c}}G_{-}(x_{0},y_{0})=I_{A_{x_{0}}^{c}}G_{+}(x_{0},y_{0})=I_{A_{x_{0}}^{c}} \Vert y_{0} \Vert $ and $I_{A_{y_{0}}^{c}}G_{-}(x_{0},y_{0})=I_{A_{y_{0}}^{c}}G_{+}(x_{0},y_{0})=0$, one knows that $M\subset A_{x_{0}}$ and that $M\subset A_{y_{0}}$.

Take an arbitrary *s* in $L^{0}({\mathcal {F}}, R)$ such that

6 $$ G_{-}(x_{0},y_{0})< s< G_{+}(x_{0},y_{0}) \quad\text{on }M. $$

Define $W=\{ry_{0}:r\in L^{0}({\mathcal {F}}, R)\}$ and $f_{s}(ry_{0})=I_{M}rs,\forall r\in L^{0}({\mathcal {F}}, R)$, then it is clear that *W* is an $L^{0}({\mathcal {F}}, R)$-submodule of *E*, that $f_{s}$ is an $L^{0}$-linear function on *W*, and that

$$f_{s}(ry_{0})=I_{M}rs\leq I_{M}rG_{+}(x_{0},y_{0})=I_{M}G_{+}(x_{0},ry_{0}),\quad \forall r\in L^{0}_{+}. $$

For any $r\in L^{0}({\mathcal {F}}, R)\backslash L^{0}_{+}$, denote $D=M\cap[r<0]$, then $f_{s}(-I_{D}ry_{0})=-I_{D}rs\geq -I_{D}rG_{-}(x_{0},y_{0})=G_{-}(x_{0},-I_{D}ry_{0})=-G_{+}(x_{0},I_{D}ry_{0})$, and hence $f_{s}(I_{D}ry_{0})\leq G_{+}(x_{0},I_{D}ry_{0})=I_{D}G_{+}(x_{0},ry_{0})$. Combining the fact that $f_{s}(I_{M\backslash D}ry_{0})=I_{M\backslash D}rs\leq I_{M\backslash D}G_{+}(x_{0},ry_{0})$, we can see that $f_{s}(ry_{0})\leq I_{D}G_{+}(x_{0},ry_{0})+I_{M\backslash D}G_{+}(x_{0},ry_{0})=I_{M}G_{+}(x_{0},ry_{0})$. Thus

$$f_{s}(ry_{0})\leq I_{M}G_{+}(x_{0},ry_{0}),\quad \forall r\in L^{0}({\mathcal {F}}, R). $$

By Theorem 2.1 there exists an $L^{0}$-linear function $x_{s}^{\ast }:E\rightarrow L^{0}({\mathcal {F}}, R)$ such that $x_{s}^{\ast}| _{W}=f_{s}$ and $x_{s}^{\ast}(y)\leq G_{+}(x_{0},y),\forall y\in E$.

Without loss of generality, suppose that *E* is a complex RN module. Let

$$g_{s}(y)=x_{s}^{\ast}(y)-ix_{s}^{\ast}(iy). $$

It is easy to show that $g_{s}:E\rightarrow L^{0}({\mathcal {F}}, C)$ is $L^{0}$-linear and $\operatorname{Re}g_{s}=x_{s}^{\ast}$, from which it follows that $\operatorname{Re}g_{s}(y)\leq G_{+}(x_{0},y),\forall y\in E$. Then we obtain

$$ \operatorname{Re}g_{s}(y)\leq G_{+}(x_{0},y)\leq\frac{ \Vert x_{0}+y \Vert - \Vert x_{0} \Vert }{1}\leq \Vert y \Vert ,\quad \forall y\in E $$

and

$$ \operatorname{Re}g_{s}(y)=-\operatorname{Re}g_{s}(-y)\geq-G_{+}(x_{0},-y)=G_{-}(x_{0},y), $$

which together with the fact that $\Vert g_{s} \Vert ^{\ast}= \Vert \operatorname{Re}g_{s} \Vert ^{\ast}$ yields that $g_{s}\in E^{\ast}$, $\Vert g_{s} \Vert ^{\ast}\leq1$ and $G_{-}(x_{0},x_{0})\leq \operatorname{Re}g_{s}(x_{0})\leq G_{+}(x_{0},x_{0})$. Thus $\operatorname{Re}g_{s}(x_{0})=I_{A_{x_{0}}}$ and so $\Vert I_{A_{x_{0}}}g_{s} \Vert ^{\ast }=I_{A_{x_{0}}}$. Namely, $I_{A_{x_{0}}}g_{s}$ supports $U(E)$ at $x_{0}$.

Therefore, for any $s_{1}$ and $s_{2}$ satisfying (6) and $s_{1}I_{M}\neq s_{2}I_{M}$, there exist two corresponding $g_{s_{1}}$ and $g_{s_{2}}$ in $E^{\ast}$ such that both $I_{A_{x_{0}}}g_{s_{1}}$ and $I_{A_{x_{0}}}g_{s_{2}}$ support $U(E)$ at $x_{0}$ and $\Vert I_{A_{x_{0}}}g_{s_{1}} \Vert ^{\ast}= \Vert I_{A_{x_{0}}}g_{s_{2}} \Vert ^{\ast}=I_{A_{x_{0}}}$. Noticing that

$$\begin{aligned} &I_{A_{x_{0}}}\operatorname{Re}g_{s_{1}}(y_{0})=I_{A_{x_{0}}}x_{s_{1}}^{\ast }(y_{0})=I_{A_{x_{0}}}f_{s_{1}}(y_{0})=I_{M}s_{1} \\ & I_{A_{x_{0}}}\operatorname{Re}g_{s_{2}}(y_{0})=I_{A_{x_{0}}}x_{s_{2}}^{\ast }(y_{0})=I_{A_{x_{0}}}f_{s_{2}}(y_{0})=I_{M}s_{2}, \end{aligned}$$

we derive that $I_{A_{x_{0}}}\operatorname{Re}g_{s_{1}}\neq I_{A_{x_{0}}}\operatorname{Re}g_{s_{2}}$, which is a contradiction to Proposition 3.6.

(⇐). Suppose that $\Vert \cdot \Vert $ is Gâteaux differentiable at $x_{0}$, *f* in $S(E^{\ast})$ supports $U(E)$ at $x_{0}$. Then it is obvious that $A_{f}\subset A_{x_{0}}$. By Lemma 4.5 we have

7 $$ I_{A_{f}}\operatorname{Re}f(y)=I_{A_{f}}G(x_{0},y),\quad \forall y\in E. $$

If $f_{1}$ and $f_{2}$ in $S(E^{\ast})$ both support $U(E)$ at $x_{0}$ and $P(A_{f_{1}f_{2}})>0$, then $I_{A_{f_{1}f_{2}}}\operatorname{Re}f_{1}=I_{A_{f_{1}f_{2}}}\operatorname{Re}f_{2}$, which implies that $I_{A_{f_{1}f_{2}}}f_{1}=I_{A_{f_{1}f_{2}}}f_{2}$. By Proposition 3.6 $x_{0}$ is a point of random smoothness of $U(E)$.

(2). It is clear by (7). □

### Remark 4.7

It should be pointed out that further development of random smoothness under Definition 3.5 confronted some obstacles, one of which is how to establish the connection between random smoothness of an RN module *E* and classical smoothness of the abstract $L^{p}$ space derived from *E*. It is an interesting problem and attracts some attention in different literature [19, 24].
